# Response of stomatal conductance, transpiration, and photosynthesis to light and CO_2_ for rice leaves with different appearance days

**DOI:** 10.3389/fpls.2024.1397948

**Published:** 2024-08-01

**Authors:** Yuping Lv, Linhui Gu, Runze Man, Xiaoyin Liu, Junzeng Xu

**Affiliations:** ^1^ College of Hydraulic Science and Engineering, Yangzhou University, Yangzhou, Jiangsu, China; ^2^ College of Agricultural Science and Engineering, Hohai University, Nanjing, Jiangsu, China

**Keywords:** photosynthetic rate, transpiration rate, stomatal conductance, light response, CO_2_ response, leaf with different appearance days

## Abstract

To investigate the dynamics of stomata, transpiration, and photosynthesis under varying light intensities and CO_2_ conditions during leaf development, the light response and CO_2_ response of stomatal conductance (*g*_sw_), transpiration rate (*T*_r_), and net photosynthetic rate (*P*_n_) were observed for rice leaves at different days after leaf emergence (DAE). The results showed that (1) as photosynthetically active radiation (PAR) increased, leaf *g*_sw_, *T*_r_, and *P*_n_ initially increased rapidly and linearly, followed by a more gradual rise to maximum values, and then either stabilized or showed a declining trend. The maximum *g*_sw_, *T*_r_, and *P*_n_ were smaller and occurred earlier for old leaves than for young leaves. The *g*_sw_, *T*_r_, and *P*_n_ all exhibited a linear decreasing trend with increasing DAE, and the rate of decrease slowed down with the reduction in PAR; (2) as the CO_2_ concentration (*C*_a_) increased, *g*_sw_ and *T*_r_ decreased gradually to a stable minimum value, while *P*_n_ increased linearly and slowly up to the maximum and then kept stable or decreased. The *g*_sw_, *T*_r_, and *P*_n_ values initially kept high and then decreased with the increase of DAE. These results contribute to understanding the dynamics in *g*_sw_, *T*_r_, and *P*_n_ during rice leaf growth and their response to varied light and CO_2_ concentration conditions and provide mechanistic support to estimate dynamic evapotranspiration and net ecosystem productivity at field-scale and a larger scale in paddy field ecosystems through the upscaling of leaf-level stomatal conductance, transpiration, and photosynthesis.

## 1 Introduction

Stomata play a crucial role in regulating water loss through transpiration and carbon dioxide (CO_2_) uptake for photosynthesis, significantly influencing water use efficiency and plant productivity (Lawson and Vialet-Chabrand, 2019). Understanding the responses of stomatal conductance (*g*_sw_), transpiration rate (*T*_r_), and net photosynthetic rates (*P*_n_) to environmental factors is essential to assess evapotranspiration and net ecosystem productivity in agroecosystems (Bellasio, 2023; Konieczna et al., 2023; Lv et al., 2024). Research into the intricate dynamics of *g*_sw_, *T*_r_, and *P*_n_ across different environments improves predictive abilities and refines strategies for water utilization and agricultural optimization, which contributes to developing sustainable agricultural strategies aimed at maximizing productivity while minimizing water consumption (Elfadl and Luukkanen, 2006; Katul, 2023; Wu et al., 2023).

Several factors, including crop canopy structure (leaf area index, leaf tilt angle, etc.), leaf nutrient elements (nitrogen, chlorophyll, etc.), soil water-thermal conditions, and meteorological factors (solar radiation, CO_2_ concentration, temperature, atmospheric humidity, etc.), have been widely studied for their influence on leaf *g*_sw_, *T*_r_, and *P*_n_ (Chen et al., 2011; Xu et al., 2015; Liu et al., 2019). The impact of light and CO_2_, as the primary energy source and substrate for plant photosynthesis, on leaf *g*_sw_, *T*_r_, and *P*_n_ have been extensively studied (Baroli et al., 2008, Li et al., 2023, Yi et al., 2023). With increased light intensity and CO_2_ concentration, leaf *P*_n_ initially increase rapidly and then slowly up to the maximum, followed by a declining trend or a stable state, which have been universally acknowledged on various crops (Kabir et al., 2023). Yu et al. (2004) reported that winter wheat *g*_sw_ decreases with increased CO_2_ concentration and increases with increased light intensity, Marín et al. (2014) stated that tobacco *T*_r_ is higher at high than at low light intensities, and Kirschbaum and McMillan (2018) showed that increasing atmospheric CO_2_ concentrations reduce canopy transpiration. Additionally, the duration (such as cumulative time, thermal time accumulation, or radiant heat accumulation) after leaf emergence also leads to changes in leaf *g*_sw_, *T*_r_, and *P*_n_ due to changes in both leaf traits (Legner et al., 2014; Scoffoni et al., 2016; Hirooka et al., 2018) and biomass sink–source relations (Kitajima et al., 2002; Xie and Luo, 2003) along with leaf aging from leaf appearance to senescence—for example, Vos and Oyarzun (1987) reported that potato *P*_n_ and *g*_sw_ decreased at near-saturating irradiance with leaf age, Echer and Rosolem (2015) stated that cotton *P*_n_ and *g*_sw_ decreased in the order of 15-, 30-, 45-, and 60-day-old leaves. Locke and Ort (2014) showed that soybean *P*_n_ decreased at a specific light intensity. However, the response of *g*_sw_, *T*_r_, and *P*_n_ to light and CO_2_, respectively, are rarely reported for rice leaves with different durations after emergence.

As the most important staple food crop in the world, the three-dimensional canopy structure of rice, describing the elongation process and spatial distribution of various organs (leaves, leaf sheaths, stems, and panicles), has been widely studied (Watanabe et al., 2005; Song et al., 2013). Temporal leaf evapotranspiration and photosynthesis with detailed 3D representation of canopy architecture are necessary to estimate seasonal variation in evapotranspiration and ecosystem productivity at field-scale and a larger scale in paddy field ecosystems, which are often achieved through the upscaling of leaf-level stomatal conductance, transpiration, or photosynthesis (Van der Zande et al., 2009; Chang et al., 2019; Shi et al., 2019). Measured light-saturated rice *P*_n_ reaches a maximum at the fully developed stage and then declines gradually as leaves senesce (Wang et al., 2009) or decreases from the top (young leaves) to the base (old leaves) within the rice canopy (Murchie et al., 2002; Jin et al., 2004). The response of *P*_n_ to light and CO_2_ also changes as rice leaves age (Xu et al., 2019). Thus, it is well known that *g*_sw_ and *T*_r_, under different light density and CO_2_ concentration conditions, also vary among leaves with various durations after leaf emergence. However, the response of *g*_sw_ and *T*_r_ to light and CO_2_ is rarely reported for rice leaves with different durations after leaf emergence.

The southern regions of the Yangtze River constitute the primary rice cultivation area in China (You et al., 2011). Understanding how the duration after leaf emergence affects *P*_n_, *g*_sw_, and *T*_r_ under different light density and CO_2_ concentration conditions is essential to unravel the physiological mechanisms of crop transpiration and photosynthesis and to assess seasonal changes in evapotranspiration and ecosystem productivity under different environmental conditions. Therefore, this study aimed to elucidate and analyze the influence of different days after leaf emergence (DAE) on *P*_n_, *g*_sw_, and *T*_r_ as well as their quantitative relationships with DAE. This will help to understand the dynamic changes in *P*_n_, *g*_sw_, and *T*_r_ and provide a reference to clarify the mechanism of transpiration and photosynthesis during the growth process of rice leaves.

## 2 Materials and methods

The Japonica Rice NJ46 was transplanted with 13 cm × 25 cm hill spacing on July 1, 2017 and harvested on October 26, 2017 in Kunshan, East China (31°15′50″ N, 120°57′43″ E) under field conditions. The rice field extended approximately 200 m in all directions. The region has a subtropical monsoon climate, with average temperature, mean relative humidity, and seasonal precipitation of 25.9°C, 76.9%, and 450.8 mm during the 2017 rice season. Irrigation, fertilizer, and pesticides were applied according to local farming practice (Guo et al., 2017; Li et al., 2023; Lv et al., 2024). To record DAE for subsequent data collection, three latest-emerged leaves on approximately 20 rice plants were tagged at 2-day intervals during tillering, jointing, and booting stages. Using a photosynthesis system (LI- 6800; LI-COR, Lincoln, NE, USA) equipped with a red/blue LED light source (LI-6800–02B) and a charged CO_2_ cartridge (CO_2_ source), the response of leaf stomatal conductance (*g*_sw_), transpiration rate (*T*_r_), and net photosynthetic rate (*P*_n_) to photosynthetically active radiation (PAR) and atmospheric CO_2_ concentration (*C*_a_) were measured for tagged leaves at various DAE values at booting and heading stages. The chamber temperature and relative humidity were set as 30°C and 70%, and the measurements were conducted under saturated soil moisture conditions at 8:00–12:00 a.m. on randomly selected sunny days during jointing and heading stages. For the response of *g*_sw_, *T*_r_, and *P*_n_ to PAR, the *C*_a_ and PAR were set at 400 μmol mol^-1^ (approximate atmospheric CO_2_ concentration) and 2,000 μmol m^-2^ s^-1^, and such a condition was maintained for 15 min for adaptation and stabilization of leaf photosynthesis prior to measurement. Then, leaf *g*_sw_, *T*_r_, and *P*_n_ were recorded automatically at 120-s intervals at 19 PAR levels (in decreasing order of 2,000, 1,950, 1,900, 1,800, 1,600, 1,400, 1,200, 1,000, 800, 600, 400, 300, 200, 150, 100, 70, 50, 30, and 0 μmol m^-2^ s^-1^). For the response of *g*_sw_, *T*_r_, and *P*_n_ to *C*_a_, the *C*_a_ and PAR were set at 400 μmol mol^-1^ and 1,600 μmol m^-2^ s^-1^ [slightly lower than saturation light intensity (Xu et al., 2019) to prevent photo inhibition], and leaf *g*_sw_, *T*_r_, and *P*_n_ were recorded automatically at 120-s intervals at 14 *C*_a_ levels (in the order of 400, 300, 200, 100, 50, 400, 400, 500, 600, 800, 1,000, 1,300, 1,500, and 1,800 μmol mol^–1^) after a 15-min pre-treatment. Totally, 37 response curves to PAR and 24 curves to *C*_a_ were measured, evenly distributed across DAE values ranging from 3 to 55.

## 3 Results

### 3.1 Light response of stomatal conductance, transpiration, and photosynthesis for rice leaves with different days after leaf emergence

**Figure 1:**
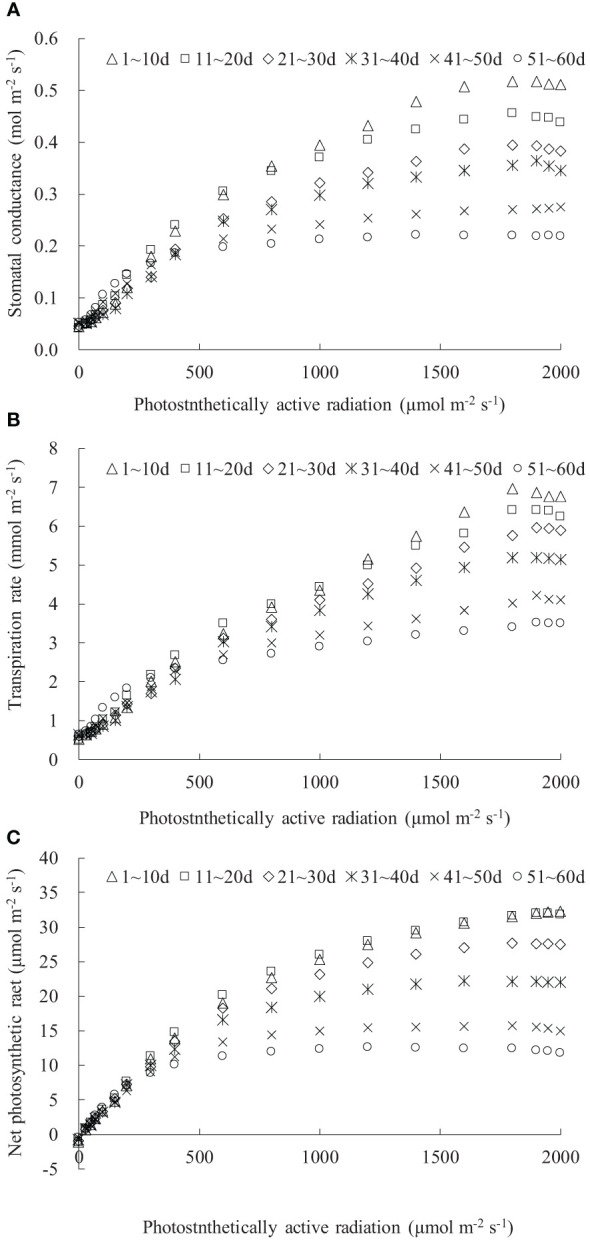
**(A–C)** The light response of stomatal conductance, transpiration rate, and net photosynthetic rate for rice leaves with different ranges of days after leaf emergence (“*m*~*n* d” in the legend indicates the days after leaf emergence; ranges from *m* to *n*).

The *g*_sw_, *T*_r_, and *P*_n_ values were influenced by both the DAE and PAR (**Figure 1**). Under dark conditions (PAR = 0 μmol m^-2^ s^-1^), leaves at different DAE maintained relatively low *g*_sw_ and *T*_r_ and negative *P*_n_. As PAR increased, *g*_sw_, *T*_r_, and *P*_n_ initially exhibited a linear and rapid increase, and then the increase rate (indicated by *dg*_sw_/*d*PAR, *dT*_r_/*d*PAR, and *dP*_n_/*d*PAR) gradually slowed down. When PAR reached a certain light intensity (referred to the saturation light intensity for *g*_sw_, *T*_r_, and *P*_n_, respectively), *g*_sw_, *T*_r_, and *P*_n_ reached their maximum values. Subsequently, with further increases in PAR, there was a declining trend (for leaves at DAE lower than approximately 40 days) or a stable state (for leaves at *DAE* higher than approximately 40 days). The *g*_sw_, *T*_r_, and *P*_n_, as well as their increase rates with increasing PAR among leaves at different DAE, exhibited similar values under low PAR conditions and showed more pronounced differences as PAR increased.

Both the maximum *g*_sw_ and its corresponding saturation light intensity decreased with increasing DAE, with older leaves reaching maximum *g*_sw_ at lower PAR conditions (**Figure 1A**). The maximum *g*_sw_ were 0.517, 0.456, 0.394, 0.364, 0.275, and 0.221 mol m^-2^ s^-1^ for leaves at *DAE* of 1–10, 11–20, 21–30, 31–40, 41–50, and 51–60 days. Young leaves (low DAE) maintained high *g*_sw_, facilitating photosynthesis and transpiration under high PAR conditions. The light response curves of leaf *T*_r_ were distinctly influenced by DAE (**Figure 1B**). The *T*_r_ at specific PAR values, as well as the saturation light intensity when *T*_r_ reached the maximum, considerably decreased with increasing DAE. The average *P*_n_ values, respectively, were -1.173, -1.141, -0.990, -0.720, -0.519, and -0.462 μmol m^-2^ s^-1^ for leaves at DAE of 1–10, 11–20, 21–30, 31–40, 41–50, and 51–60 days under PAR = 0 μmol m^-2^ s^-1^ conditions (**Figure 1C**). The negative *P*_n_ observed under no-light conditions represented leaf respiration capacity. The decreased absolute value of *P*_n_ with increasing DAE implied that the leaf respiration rate attenuated with an increase in DAE, attributable to the exuberant leaf respiration for young leaves. The maximum *P*_n_ values were 32.263, 31.959, 27.645, 22.164, 15.676, and 12.582 μmol m^-2^ s^-1^, respectively, for leaves at DAE of 1–10, 11–20, 21–30, 31–40, 41–50, and 51–60 days. Young leaves sustained high *P*_n_ under high PAR conditions and exhibited vigorous physiological growth.

**Figure 2:**
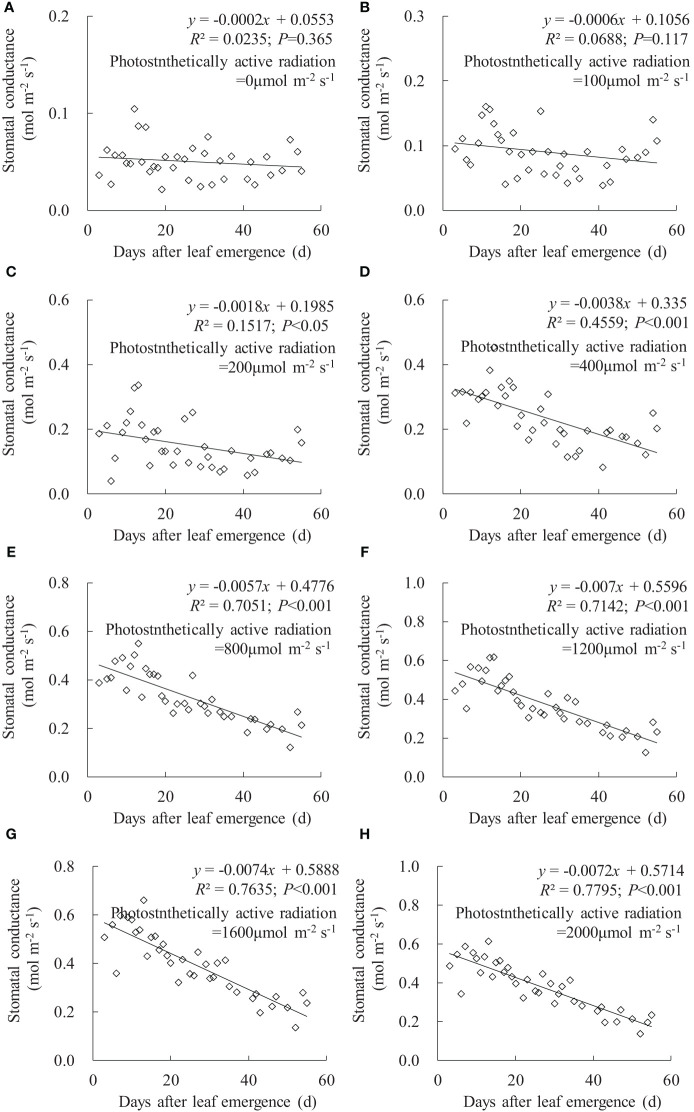
**(A–H)** Impact of days after leaf emergence on stomatal conductance under different photosynthetically active radiation conditions.

In any PAR condition, leaf *g*_sw_ linearly decreased with an increase in DAE, and the decrease rate (indicated by the absolute value of the slope of the linear regression line) increased with enhanced PAR (**Figure 2**). Under PAR of 0 and 100 μmol m^-2^ s^-1^ conditions, DAE had a negligible impact on leaf *g*_sw_, and the leaves consistently maintained a lower *g*_sw_ value. Under PAR of 200, 400, and 800 μmol m^-2^ s^-1^ conditions, the slopes of *g*_sw_ against DAE were -0.0018, -0.0038, and -0.0057, respectively; the leaf *g*_sw_ significantly decreased with increasing DAE, and there are noticeable differences in both leaf *g*_sw_ and the decrease rate among different PAR intensities. Under conditions of PAR higher than 1,200 μmol m^-2^ s^-1^, the decrease rate in leaf *g*_sw_ with DAE was approximately 0.007, and leaf *g*_sw_ ranged from 0.127 to 0.659 mmol m^-2^ s^-1^. Leaf *g*_sw_ significantly decreased with increasing DAE, but the differences in both leaf *g*_sw_ and the decrease rate were less pronounced among different *PAR* intensities.

**Figure 3:**
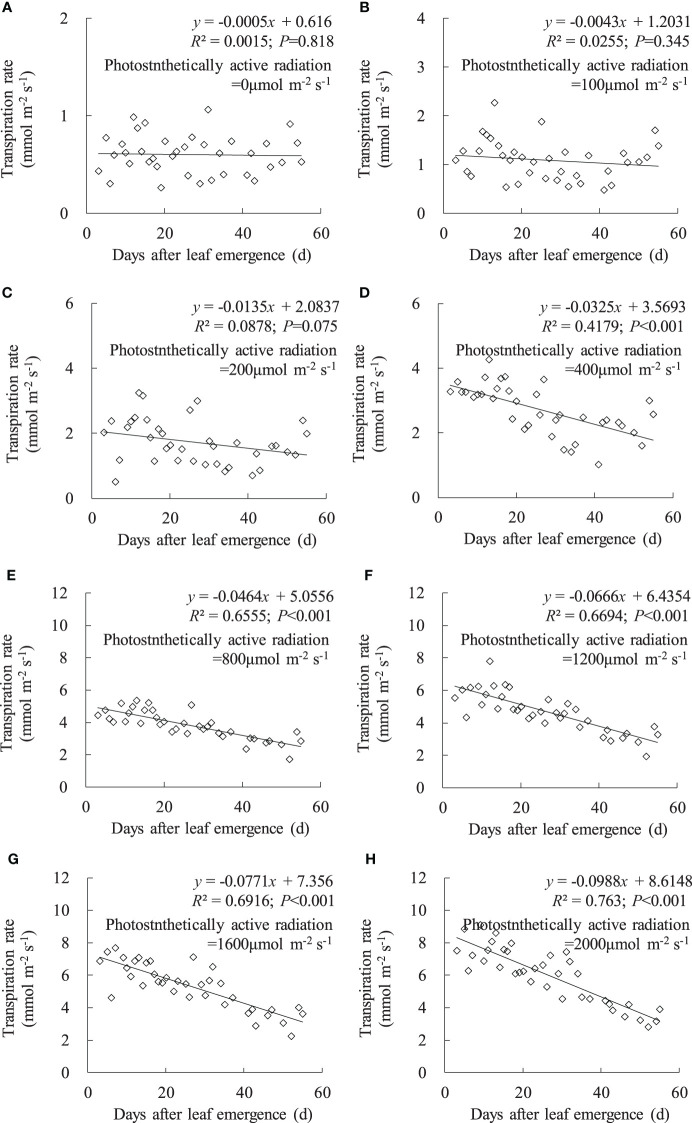
**(A–H)** Impact of days after leaf emergence on transpiration rate under different photosynthetically active radiation conditions.

Consistent with the variation in leaf *g*_sw_, leaf *T*_r_ linearly decreased with an increase in DAE under any PAR condition, and the decrease rate increased with enhanced PAR (**Figure 3**). Under PAR conditions lower than 200 μmol m^-2^ s^-1^, leaf *T*_r_ external environmental demand for leaf evaporation is weak, and leaf *T*_r_ remains consistently low, with no significant decrease in leaf *T*_r_ with increasing DAE. Under PAR intensities of 400, 800, 1,200, 1,600, and 2,000 μmol m^-2^ s^-1^, leaf *T*_r_ respectively ranged from 1.015 to 4.265, 1.724 to 5.359, 1.938 to 7.790, 2.221 to 7.677, and 2.819 to 9.072 mmol m^-2^ s^-1^, and the slopes of leaf *T*_r_ against DAE, respectively, were -0.0325, -0.0464, -0.0666, -0.0771, and -0.0988. Under high PAR conditions (exceeding 400 μmol m^-2^ s^-1^), leaf *T*_r_ significantly decreased, and the decrease rate becomes more pronounced with increasing PAR. Younger leaves can maintain higher *T*_r_ under high light conditions to expedite transpirational cooling, enabling the leaves to remain within the optimal temperature range for physiological activities. As the leaves aged, physiological activity decreased, and leaf adaptability to light intensity decreased, resulting in lower *T*_r_ under high light conditions.

**Figure 4:**
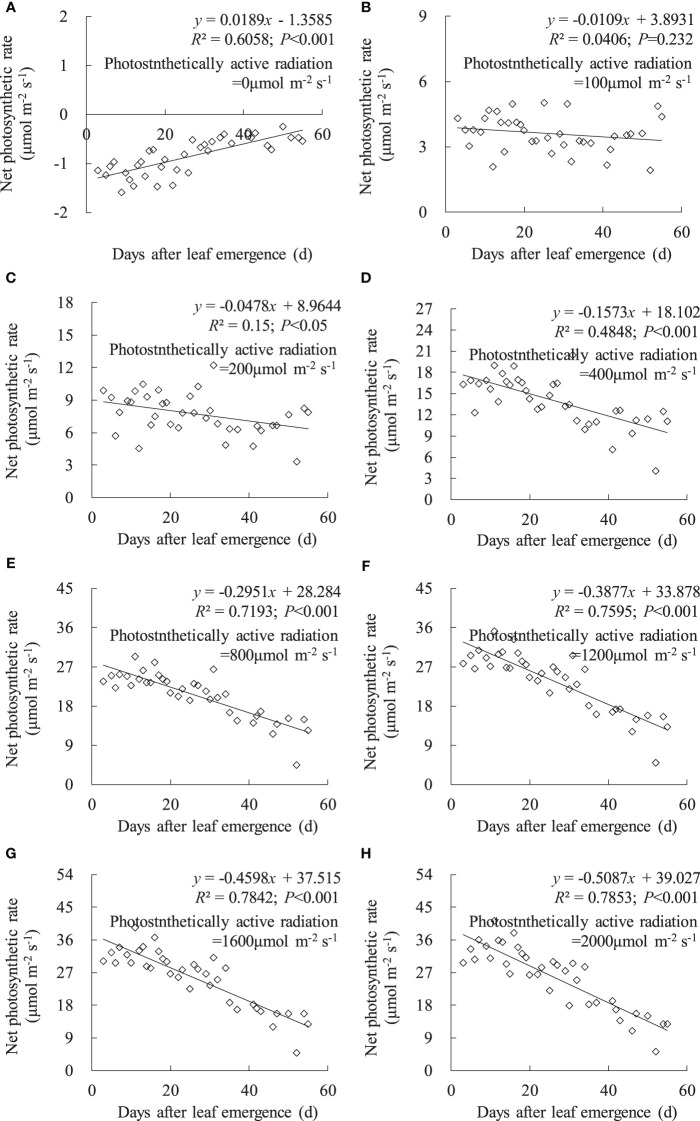
**(A–H)** Impact of days after leaf emergence on net photosynthetic rate under different photosynthetically active radiation conditions.

Under no-light conditions (PAR = 0 μmol m^-2^ s^-1^), leaf *P*_n_ was negative, and *P*_n_ linearly increased with DAE (**Figure 4**). The leaves were unable to perform photosynthesis under zero light intensity, and leaves with low DAE exhibited a stronger metabolic activity, reflected in a higher respiration rate (manifested as negative values). Under a PAR of 100 μmol m^-2^ s^-1^, leaf *P*_n_ remained at approximately 3.6 μmol m^-2^ s^-1^, with no significant change in leaf *P*_n_ with increasing DAE. Under PAR conditions higher than 200 μmol m^-2^ s^-1^, leaf *P*_n_ significantly decreased with increasing DAE, and the magnitude of decrease became more pronounced with enhanced PAR.

### 3.2 CO_2_ response of stomatal conductance, transpiration and photosynthesis for rice leaves with different days after emergence

**Figure 5:**
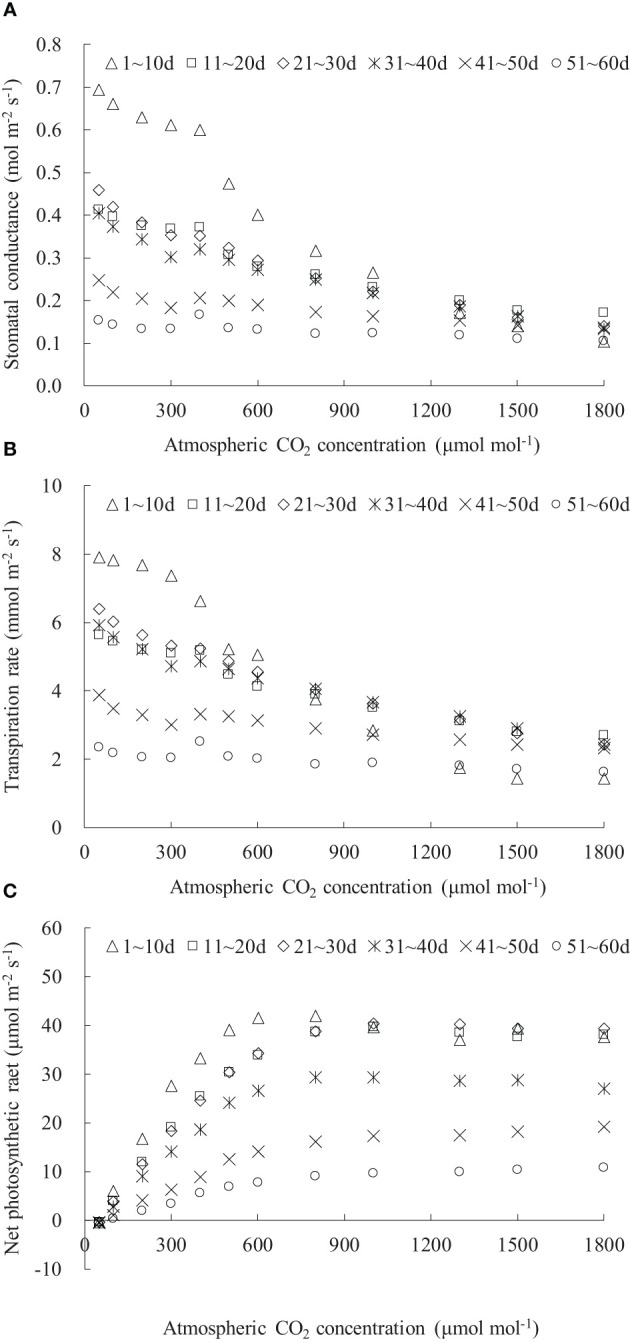
**(A–C)** CO_2_ response of stomatal conductance, transpiration rate, and net photosynthetic rate of rice leaves with different ranges of days after leaf emergence (“*m*~*n* d” in the legend indicates the days after leaf emergence; ranges from *m* to *n*).

The *C*_a_ considerably influenced *g*_sw_, *T*_r_, and *P*_n_ in rice leaves (**Figure 5**). The diffusion of CO_2_ from the outside to the inside of the leaf primarily relied on stomata; an increase in *C*_a_ led to a reduction in leaf *g*_sw_, followed by a decrease in leaf *T*_r_ (**Figures 5A, B**). Leaf *g*_sw_ and *T*_r_ gradually decreased with increasing *C*_a_ and DAE, and their decreasing rate slowed down as *C*_a_ increased. When *C*_a_ increased to approximately 1,500 μmol mol^-1^, leaf *g*_sw_ and *T*_r_ stabilized at the minimum values. Under the *C*_a_ range of 0 to 1,800 μmol mol^-1^, leaf *g*_sw_ respectively ranged from 0.103 to 0.693, 0.171 to 0.411, 0.139 to 0.458, 0.133 to 0.404, 0.135 to 0.247, and 0.104 to 0.165 mol m^-2^ s^-1^, and leaf *T*_r_ respectively ranged from 1.426 to 7.895, 2.694 to 5.622, 2.431 to 6.401, 2.423 to 5.912, 2.326 to 3.872, and 1.615 to 2.514 mmol m^-2^ s^-1^ for DAE of 1–10, 11–20, 21–30, 31–40, 41–50, and 51–60 days. Both leaf *g*_sw_ and *T*_r_ decreased with increasing DAE under specific *C*_a_ conditions. Leaves at smaller DAE maintained higher *g*_sw_ and *T*_r_ at low *C*_a_, indicating that vigorously growing leaves sustained higher *g*_sw_ for physiological processes (such as transpiration and photosynthesis) and exhibited robust physiological activity even under low *C*_a_ conditions. There was a relatively small difference in leaf *g*_sw_ and *T*_r_ among leaves at different DAE at high *C*_a_ concentrations. Leaves with larger DAE (41–50 and 51–60 days) showed limited sensitivity of *g*_sw_ and *T*_r_ to changes in *C*_a_ concentration, maintaining consistently lower values regardless of the variations in *C*_a_ concentration.

The leaf *P*_n_ at different DAE exhibited a similar trend with changing atmospheric *C*_a_ (**Figure 5C**). The increase rate of leaf *P*_n_ (indicated by *dP*_n_/*dC*_a_) gradually slowed down with increasing DAE. As *C*_a_ increased, rice leaf *P*_n_ initially increased rapidly in a linear fashion, and the increase rate subsequently decreased, and leaf *P*_n_ gradually reached its maximum value, resulting in either a stable or a declining *P*_n_. Under CO_2_ concentrations lower than 50 μmol mol^-1^, leaf photosynthesis was constrained by the available CO_2_ concentration; larger stomatal conductance could not compensate for the impact of low CO_2_ concentration, resulting in lower leaf photosynthesis than respiration, leading to CO_2_ emission (negative *P*_n_ values). Within the *C*_a_ range of 0 to 1,800 μmol mol^-1^, leaf *P*_n_ ranged from -0.437 to 41.866, -0.419 to 39.614, -0.491 to 40.345, -0.639 to 29.344, -0.485 to 19.135, and -0.504 to 10.657 μmol m^-2^ s^-1^ for DAE of 1–10, 11–20, 21–30, 31–40, 41–50, and 51–60 days, respectively. As DAE decreases, both the peak value of *P*_n_ and the carboxylation rate (the slope of the linear segment) increased, indicating that leaves with smaller DAE possessed a stronger photosynthetic capability.

**Figure 6:**
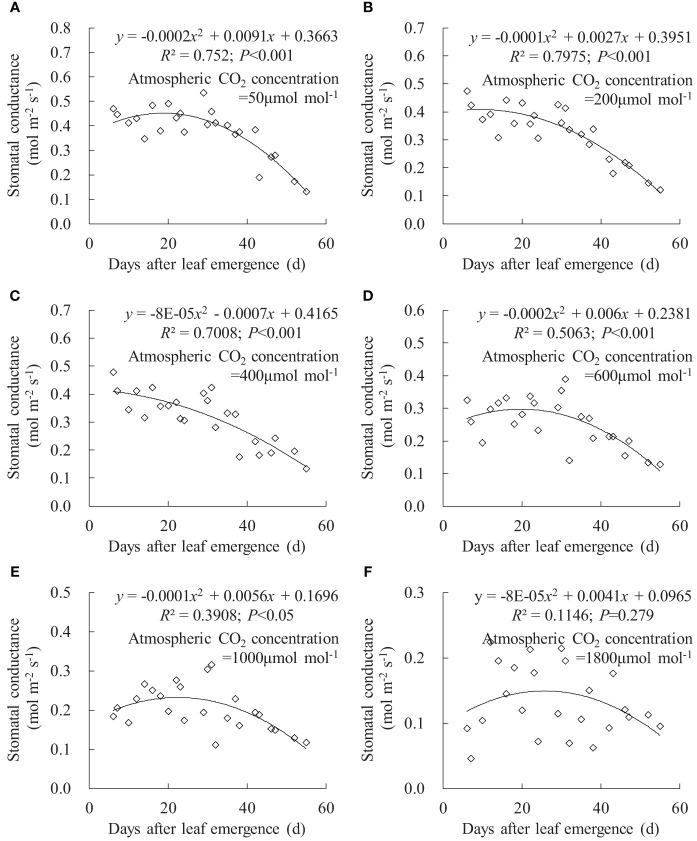
**(A–F)** Impact of days after leaf emergence on stomatal conductance under different atmospheric CO_2_ concentration conditions.

**Figure 7:**
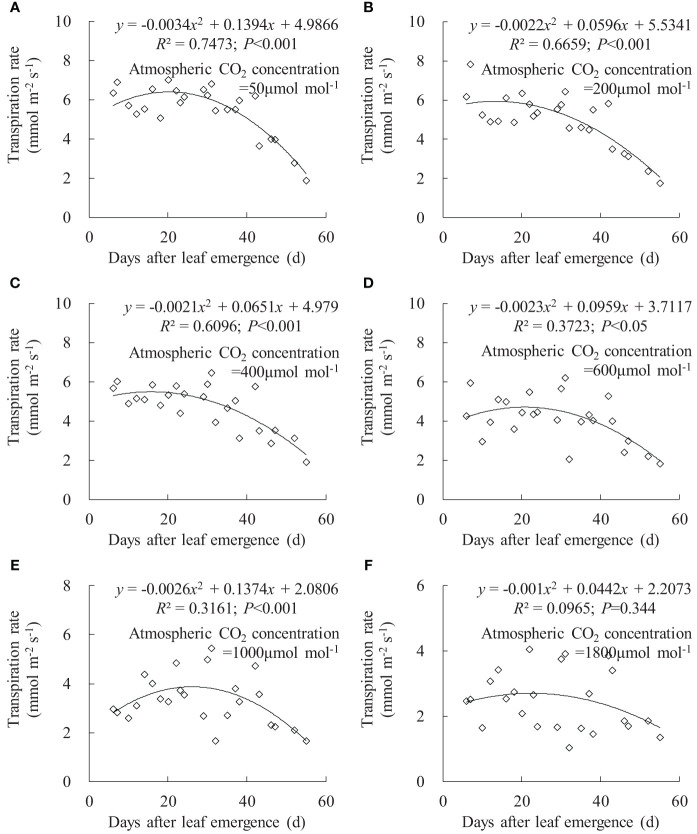
**(A–F)** Impact of days after leaf emergence on transpiration rate under different atmospheric CO_2_ concentration conditions.

**Figure 8:**
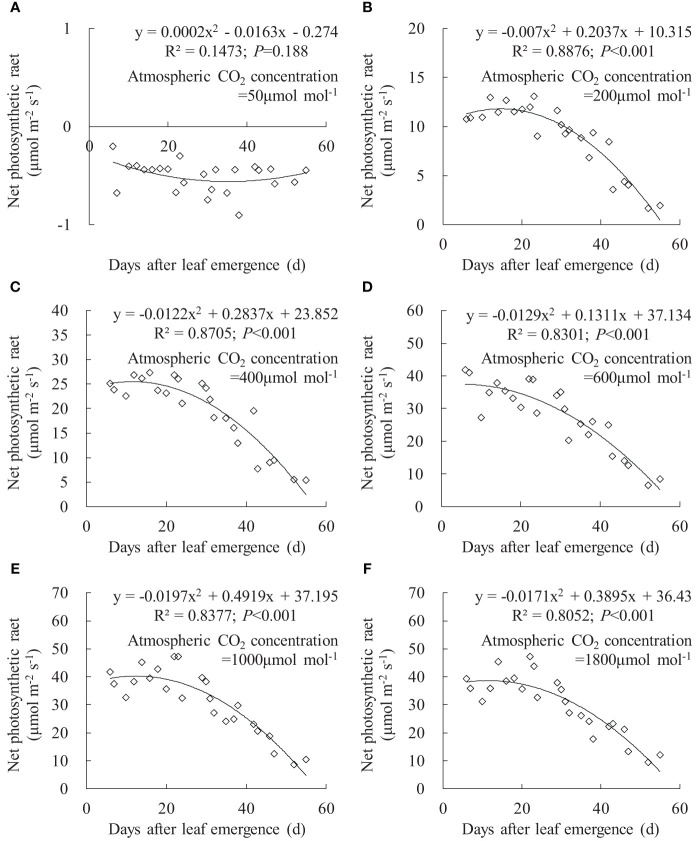
**(A–F)** Impact of days after leaf emergence on net photosynthetic rate under different atmospheric CO_2_ concentration conditions.

The relationships between *g*_sw_, *T*_r_, and *P*_n_ and DAE could be fitted using quadratic regression equations (**Figures 6**–**8**). Under *C*_a_ of 50, 200, 400, 600, 1,000, and 1,800 μmol mol^-1^, leaf *g*_sw_ respectively ranged from 0.132 to 0.535, 0.122 to 0.474, 0.134 to 0.478, 0.129 to 0.390, 0.111 to 0.316, and 0.046 to 0.224 mmol m^-2^ s^-1^. Leaf *g*_sw_ decreased along with increasing *C*_a_. As DAE increased, leaf *g*_sw_ initially remained at higher values and subsequently gradually decreased. Young leaves (small DAE) maintained higher *g*_sw_ to facilitate physiological activities at low *C*_a_ conditions. High CO_2_ concentrations (especially at a *C*_a_ of 1,800 μmol mol^-1^) inhibited stomatal aperture, and the leaf *g*_sw_ at different DAE consistently remained at lower values.

Leaf *T*_r_ exhibited a similar trend to leaf *g*_sw_ (**Figure 7**). Under *C*_a_ of 50, 200, 400, and 600 μmol mol^-1^, leaf *T*_r_ for different DAE respectively ranged from 1.876 to 7.007, 1.743 to 7.810, 1.905 to 6.467, and 1.815 to 6.202 mmol m^-2^ s^-1^. Leaf *T*_r_ decreased with increasing *C*_a_, and rice leaves at small DAE maintained a higher *T*_r_ at low *C*_a_. As DAE increased, leaf *T*_r_ initially remained at higher values and then gradually decreased. At *C*_a_ of 1,000 and 1,800 μmol mol^-1^, the impact of DAE on *T*_r_ diminished, and high CO_2_ concentration inhibited stomatal aperture and transpiration.

The variation in leaf *P*_n_ with DAE under different *C*_a_ is depicted in **Figure 8**. At *C*_a_ of 50 μmol mol^-1^, the leaf *P*_n_ at different DAE consistently remained at approximately -0.5 μmol m^-1^ s^-1^. This is primarily attributed to the limitation of photosynthetic capacity by low CO_2_ concentrations, where leaf respiration exceeded photosynthesis, resulting in CO_2_ release. At *C*_a_ of 200, 400, 600, 1,000, and 1,800 μmol mol^-1^, leaf *P*_n_ respectively ranged from 1.690 to 13.114, 5.484 to 27.375, 6.694 to 41.858, 8.576 to 47.116, and 9.304 to 47.137. Leaf *P*_n_ rapidly increased with rising *C*_a_, reaching its peak at approximately 1,000 μmol mol^-1^ *C*_a_, with no considerable difference between 1,000 and 1,800 μmol mol^-1^ *C*_a_. When *C*_a_ exceeded 200 μmol mol^-1^, leaf *P*_n_ remained relatively high at smaller DAE and gradually decreased with further increases in DAE. This indicated that vigorously growing leaves exhibited higher *P*_n_, and leaf photosynthetic capacity decreased as leaves age, leading to a decline in carbon assimilation.

## 4 Discussion

### 4.1 Effect of days after leaf emergence on the light response

As PAR was enhanced, leaf *g*_sw_, *T*_r_, and *P*_n_ initially exhibited a linear and rapid increase, followed by a gradual slowdown in the increase rate, eventually reaching a maximum value and then stabilizing or slightly decreasing thereafter (**Figure 1**). Similar trends have been observed in the flag leaves of winter wheat (Inoue et al., 2004; Carmo-Silva et al., 2017). Under no-light conditions *(PAR* = 0 μmol m^-2^ s^-1^), the leaves were unable to undergo photosynthesis, resulting in metabolic CO_2_ emission (with leaf *P*_n_ showing as a negative value). Leaves at smaller DAE released more CO_2_ due to their vigorous metabolic activity (Pantin et al., 2012). Under low-light conditions, limited atmospheric evaporative capacity and insufficient PAR for photosynthesis led to lower *g*_sw_, *T*_r_, and *P*_n_ regardless of the variations in DAE. As PAR intensified, leaf stomatal opening widened, leading to an increase in *g*_sw_. Larger stomatal apertures allowed a greater influx of CO_2_ (providing an ample supply for leaf photosynthesis) and output of water vapor through the stomata; thus, leaf *P*_n_ and *T*_r_ increased. Simultaneously, the increased atmospheric evaporative capacity caused by enhanced PAR also resulted in higher *T*_r_. Leaves with larger DAE reached the light saturation point earlier, and *g*_sw_, *T*_r_, and *P*_n_, under saturated light conditions, decreased with increasing DAE, suggesting that young leaves could maintain larger stomatal apertures for efficient transpiration and photosynthesis under high light intensity (high *T*_r_ and *P*_n_). As the leaves aged, their adaptation to high light weakened, and leaves with larger DAE could not fully utilize high light intensity for photosynthesis.

Under a specific PAR condition, *g*_sw_, *T*_r_, and *P*_n_ showed a consistent linear decrement with the increase in DAE (**Figures 2**–**4**). This finding was congruent with the decline in *g*_sw_ and *P*_n_ with potato leaf senesced (Vos and Oyarzun, 1987). Echer and Rosolem (2015) also asserted that cotton leaf DAE had nominal impact on leaf *P*_n_ under low PAR, while *P*_n_ was notably higher in 15- and 30-day-old leaves compared to 45- and 60-day-old leaves when PAR exceeded a threshold, with both *T*_r_ and *g*_sw_ reduced as the leaves aged and the light intensity waned. Hossain et al. (2007) and Jin et al. (2004) reported that rice *g*_sw_, *T*_r_, and *P*_n_, at particular PAR, decreased significantly with lowering leaf position, which was consistent with the current research, as newly emerged rice leaves appeared in the upper canopy, implicating a reduction in leaf DAE as leaf position decreased. Generally, rice leaf photosynthesis was highly related to leaf nitrogen level, efficiencies of radiant energy utilization, electron transport, and photophosphorylation, and these values decreased with leaf aging (or downward leaves) (Murchie et al., 2002; Suzuki et al., 2009; Okami et al., 2016; Yang et al., 2016), which also agreed with the decreased *P*_n_. In contrast, Wang et al. (2009) reported that the measured light-saturated rice *g*_sw_, *T*_r_, and *P*_n_ reached the maximum at the last second fully developed leaf and then declined gradually in downward leaves at nine-leaf age (an indicator representing the developmental progress of plants) stage (tillering stage correspondingly). Xu et al. (2019) also stated that light-saturated rice *P*_n_ peaked at around 10 days after leaf emergence and then decreased as leaves aged. The discrepancy with the current study might be attributed to low-frequency measurement for photosynthetic characteristics under smaller DAE, as the measurement was inconvenient due to the small leaf area before they were fully expanded. Additionally, the measurement in the current study began at DAE of 3 days, at which time the leaves had a large leaf area. Consequently, the study did not monitor the increase in leaf *g*_sw_, *T*_r_, and *P*_n_ during the leaf expansion process.

### 4.2 Effect of days after leaf emergence on the CO_2_ response

As *C*_a_ increased, leaf *g*_sw_ and *T*_r_ gradually decreased, while *P*_n_ increased linearly and rapidly, and the amplitude of variations in *g*_sw_, *T*_r_, and *P*_n_ decelerated, eventually leading to a stabilization of minimal *g*_sw_ and *T*_r_ and an elevation of *P*_n_ to its peak, subsequently maintaining stability or experiencing a slight decline (**Figure 5**). Yasutake et al. (2016) found that 1,000 μmol mol^-1^ *C*_a_ significantly increased sweet pepper *P*_n_ but decreased *g*_sw_ and *T*_r_ compared with 400 *C*_a_. Ahmed et al. (2022) reported that *P*_n_ increased and *g*_sw_ and *T*_r_ decreased in the order of 500, 1,000, and 1,500 μmol mol^-1^ *C*_a_. This was consistent with the decreased *g*_sw_ and *T*_r_ and increased *P*_n_ with leaf aging observed in the current study. Inamoto et al. (2022) showed that the increase in Oriental Hybrid Lily *P*_n_ was greater in the low *C*_a_ range (380 to 1,000 μmol mol^-1^) and lower in the high *C*_a_ range (1,000 to 2,000 μmol mol^-1^), which agreed with the amplitude of variations in *P*_n_.

Under specific *C*_a_, the leaf *g*_sw_, *T*_r_, and *P*_n_ remained at a relatively high level when DAE was less than approximately 25 days and then gradually decreased with the further increase in DAE (**Figures 6**, **7**, **8**). Chlorophyll activity, Rubisco activity, RuBP regeneration capacity, and nitrogen content (positively correlated with the photosynthetic potential of leaves) generally exhibited an increasing trend during the leaf expansion phase, followed by the maintenance of relatively high values, and then decreased as the leaves aged (Murchie et al., 2002; Suzuki et al., 2009; Gunasekera et al., 2013), which might be the primary reasons for the variation in *g*_sw_, *T*_r_, and *P*_n_. At lower *C*_a_ concentrations, leaves at a smaller DAE maintained higher *g*_sw_ to facilitate atmospheric CO_2_ entering for transpiration and photosynthesis. Leaves at a greater DAE had weaker adaptability to external environments, maintaining lower levels of *g*_sw_, *T*_r_, and *P*_n_ regardless of *C*_a_ level. When *C*_a_ exceeded a certain level, *C*_a_ exerted a suppressive effect on stomatal conductance to reduce transpiration, but the photosynthetic rate did not decrease.

## 5 Conclusions

This study investigated the dynamics of stomatal conductance *g*_sw_, transpiration rate *T*_r_, and net photosynthetic rate *P*_n_ in rice leaves across varying light intensities and CO_2_ conditions during leaf development. The key conclusions drawn from the findings are as follows:

(1) Response to photosynthetically active radiation PAR: Increasing PAR led to an initial rapid and linear increase in *g*_sw_, *T*_r_, and *P*_n_, followed by a more gradual rise to maximum values, with subsequent stabilization or decline. Notably, old leaves reached their maximum *g*_sw_, *T*_r_, and *P*_n_ earlier and at smaller magnitudes compared to young leaves. Additionally, a linear decreasing trend in *g*_sw_, *T*_r_, and *P*_n_ with increasing DAE was observed, with the decrease rate slowing down with reduced PAR.
(2) Response to atmospheric CO_2_ concentrations C_a_: With increasing *C*_a_, *g*_sw_ and *T*_r_ decreased gradually to a stable minimum value, while *P*_n_ exhibited a linear and slow increase up to a maximum before stabilizing or decreasing. Under specific *C*_a_ conditions, rice leaf *g*_sw_, *T*_r_, and *P*_n_ initially remain at higher values and then gradually decrease with increasing DAE.

These conclusions provided crucial mechanistic insights to estimate dynamic evapotranspiration and net ecosystem productivity at both field-scale and larger scales in paddy field ecosystems by upscaling leaf-level physiological processes. This knowledge can inform more accurate predictions and management strategies to optimize agricultural practices and enhance the sustainability of rice cultivation amidst changing environmental conditions.

## Data availability statement

The datasets presented in this article are not readily available because the authors do not have permission to share data. Requests to access the datasets should be directed to YL, lvyuping@yzu.edu.cn.

## Author contributions

YL: Conceptualization, Funding acquisition, Writing – original draft. LG: Investigation, Writing – original draft. RM: Formal analysis, Validation, Writing – review & editing. XL: Writing – review & editing. JX: Writing – review & editing.
